# Transfer of energy pathway genes in microbial enhanced biological phosphorus removal communities

**DOI:** 10.1186/s12864-015-1752-5

**Published:** 2015-07-16

**Authors:** Dennis H.-J. Wong, Robert G. Beiko

**Affiliations:** Faculty of Graduate Studies, Dalhousie University, Halifax, Nova Scotia Canada; Faculty of Computer Science, Dalhousie University, Halifax, Nova Scotia Canada

**Keywords:** Enhanced biological phosphorus removal, Lateral gene transfer, Metagenomics, Phylogenetics, Mobile genetic elements

## Abstract

**Background:**

Lateral gene transfer (LGT) is an important evolutionary process in microbial evolution. In sewage treatment plants, LGT of antibiotic resistance and xenobiotic degradation-related proteins has been suggested, but the role of LGT outside these processes is unknown. Microbial communities involved in Enhanced Biological Phosphorus Removal (EBPR) have been used to treat wastewater in the last 50 years and may provide insights into adaptation to an engineered environment. We introduce two different types of analysis to identify LGT in EBPR sewage communities, based on identifying assembled sequences with more than one strong taxonomic match, and on unusual phylogenetic patterns. We applied these methods to investigate the role of LGT in six energy-related metabolic pathways.

**Results:**

The analyses identified overlapping but non-identical sets of transferred enzymes. All of these were homologous with sequences from known mobile genetic elements, and many were also in close proximity to transposases and integrases in the EBPR data set. The taxonomic method had higher sensitivity than the phylogenetic method, identifying more potential LGTs. Both analyses identified the putative transfer of five enzymes within an Australian community, two in a Danish community, and none in a US-derived culture.

**Conclusions:**

Our methods were able to identify sequences with unusual phylogenetic or compositional properties as candidate LGT events. The association of these candidates with known mobile elements supports the hypothesis of transfer. The results of our analysis strongly suggest that LGT has influenced the development of functionally important energy-related pathways in EBPR systems, but transfers may be unique to each community due to different operating conditions or taxonomic composition.

**Electronic supplementary material:**

The online version of this article (doi:10.1186/s12864-015-1752-5) contains supplementary material, which is available to authorized users.

## Background

Enhanced biological phosphorus removal (EBPR) communities are a common form of microbial treatment developed by Banard [1] that removes phosphorus and occasionally nitrogen from sewage. EBPR is environmentally sustainable and affordable [2], with microbial communities typically seeded from the local environment or from a seed stock. Considerable effort has been put into understanding EBPR, from community diversity (e.g. [3–5]), to metabolic function (e.g. [2, 6]) and engineering (e.g. [7, 8]), with the objective of improving efficiency and stability. A substantial amount of work has gone into understanding what organisms are present in EBPR plants [4, 5, 9–11], which organisms tend to be associated with each other (e.g. [4, 5]), their ecology (e.g. [10, 12, 13]), and how to engineer the EBPR process (e.g. [11, 14]). Recently, a conceptual ecosystem model [15] and a core microbiome [5] have been proposed, based mainly on 25 plants in Denmark, revealing a taxonomically broad group of characterized and uncharacterized organisms. However, the majority of EBPR-associated organisms are not found in all EBPR samples.

To develop the EBPR process, a carbon source, typically acetate or propionate, is input to the system, and anaerobic and aerobic conditions are cycled in a bioreactor to select for phosphate accumulating organisms (PAOs). Other organisms perform functions such as fermentation and hydrolysis, and are often referred to as the “flanking community” (e.g. [5, 16]). Phosphorus uptake occurs during the anaerobic cycle, and carbon- and energy-providing polymers are stored as polyhydroxyalkanoates (PHAs). During the aerobic phase, energy stored in the PHAs is used for growth and reproduction. The type of input carbon source is taken up at different rates for different organisms in EBPR, which could affect treatment plant operation [17]. Because anaerobic and aerobic cycling is so important for EBPR community function, emphasis has been placed on metabolic pathways related to PHA metabolism (e.g. [17, 18]), glycolysis and gluconeogenesis (e.g. [16, 17, 19]), the pentose phosphate pathway (e.g. [2, 20]), and the citric acid cycle (e.g. [2, 16, 19, 20]) as a means to understand how EBPR functions, and through usage of a particular metabolic pathway, a way to make EBPR more efficient at removing phosphate (e.g. [19]).

Metagenomic sequencing of two lab-scale EBPR enrichment reactors allowed the elucidation of EBPR-relevant metabolism of a major PAO, the Betaproteobacterium *Candidatus* Accumulibacter phosphatis (CAP) Clade IIA strain UW-1 [16], including phosphate uptake and PHA degradation during the aerobic phase, and PHA storage and polyphosphate degradation during the anaerobic stage. The amount of sequence generated, and the technology used (Sanger sequencing, which generates relatively long reads) allowed the eventual assembly of the first complete genome of CAP Clade IIA strain UW-1. Recently, draft genome sequences of other CAP have been obtained [21, 22]. Sequencing of a full-scale reactor metagenome from Denmark highlighted an enrichment of genes associated with biofilm and phosphate metabolism, and the taxonomic diversity of full-scale reactor communities [23]. Despite the existence of a group of organisms considered to be common in EBPR communities, the community structure and specific strains present can vary considerably between treatment plants [4].

Lateral gene transfer (LGT) is a well-established mode of evolution in bacteria that can be studied through a variety of approaches using genome sequences (e.g. [24–28]). LGT plays an important role in adaptation, for example, in heavy-metal metabolism (e.g. [29, 30]), and in antibiotic resistance (e.g. [29, 31]). Transfers tend to take place between close relatives, but many examples of transfer between more distant relatives have been reported as well (e.g. [24, 32]). LGT is known to have occurred in sewage treatment plants, impacting antibiotic resistance genes (e.g. [29, 33–36]), and xenobiotic degradation (e.g. [37, 38]). Many of these transfers are mediated by mobile genetic elements (MGEs) such as plasmids and transposons (e.g. [37, 38]). Engineering of treatment plants have used plasmids to bioaugment communities to allow metabolism of xenobiotics [39]. Other mechanisms of LGT exist, such as gene transfer agents (e.g. [40]) and transformation (e.g. [41]), but their role in sewage treatment communities is not known.

The metagenomes of two non-EBPR sludge community plasmids were sequenced [29], revealing substantial differences in genes from a plant with primarily industrial waste and a plant with primarily household waste. The differences suggested that the prominence of carbohydrate metabolism genes from the industrial waste plant, and the genes related to defense factors in the household waste plant, were the result of selection in each of those communities. Others have noted that transferred plasmids in non-EBPR sludge can have a mosaic of functional genes [33]. Some evidence of LGT has been identified in PAO genomes [21] but no such events have been proposed from metagenome data thus far.

There are many different bioinformatic approaches for the identification of LGT events (reviewed in [27, 42]), but most rely on whole-genome sequences. Different methods can identify very different sets of genes as putatively acquired via LGT (e,g, [28, 43, 44]). Metagenomic data introduce several challenges that make identification of LGT difficult, in particular, metagenome sequence fragments are short (typically < 1000 nucleotides in length) and of uncertain provenance in the community. Incorrectly assembled chimeric contigs often combine sequences from multiple members of the same genus, species or strain [45–47]. Chimeric contigs are more common in more diverse communities (e.g. [46]), and when using short-read sequencing technology with closely related strains [45], and can often lead to incorrect classification of contigs.

Despite these challenges, it would be an important step to develop sequence-based approaches to identify LGT within an environment to further our understanding of microbial adaptation. Approaches such as genetic exchange networks [48] could identify transfers between multiple taxonomic groups. Here we develop and apply two different analyses to identify candidate LGT events in EBPR metagenomic data for six relevant metabolic pathways. We focus on class-level gene transfers to avoid any errors in assembly at lower taxonomic levels that can affect the accuracy of classification. Our first method, classification discordance, exploits disagreement between taxonomic classifications of genes and longer assemblies. Our second method relies on phylogenetic incongruence. Both are then filtered by homology with known MGEs to identify putative cases of LGT that have been putatively transferred through MGEs.

## Methods

### Sequence data

The EBPR enrichment culture metagenomes for lab-scale bioreactors in Madison, Wisconsin, United States of America (USA) and Brisbane, Australia (AU) that comprised the first EBPR metagenome study [16], both sequenced using Sanger sequencing, were downloaded on April 21^st^ 2009 from the Joint Genome Institute (http://genome.jgi.doe.gov/OZEBPRsludge/OZEBPRsludge.download.htm, projectID=201007& metagenome=4463936.3). The USA community is composed of 15,866 contigs and assemblies, 25,312,906 nucleotides, with reads an average of 986 nucleotides in length, and the AU community 11,188 contigs and 24,385,629 nucleotides, with reads an average of 1038 nucleotides in length. The EBPR metagenome for a full-scale bioreactor in Aalborg, Denmark (DK) that performs nitrogen removal in addition to phosphate removal [23], sequenced using Illumina GAII (2 x 72 paired end), was downloaded from the SEED (http://metagenomics.anl.gov/metagenomics.cgi?page=MetagenomeOverview& metagenome=4463936.3), and is composed of 269,385 contigs and 145,725,513 nucleotides of sequence data. We used the assemblies and predicted genes and putative proteins as generated by the original sequencing projects.

Mobile genetic element sequence data consisted of MGEs from the Phast [49] and the ACLAME databases [50]. The Phast database is composed primarily of viral sequences and the ACLAME database is composed of plasmids, phage genomes and transposons. We also included the complete NCBI plasmid database, and added other plasmids from NCBI that were not in the plasmid database, but matched the search terms “sewage treatment”, “waste-water” and “wastewater”. In total, this amalgamated database contained 7,584,934 sequences.

### Taxonomic and functional annotation of metagenomic contigs

Class-level taxonomic classification of contigs was done using RITA [51]. RITA uses a reference database to assign a taxonomic classification to sequence data using both homology and nucleotide composition. We used RITA v1.0.1 with a reference data set of over 2986 genomes representing 65 different taxonomic classes (Additional file 1), using USEARCH v4.1.93 [52] for homology searches and FCP v1.0.3 [53] for nucleotide composition matching. RITA performs taxonomic classification and assigns sequences to one of four confidence groups based on the strength of evidence in favor of that classification. Sequences with identical taxonomic predictions from both homology and composition were assigned to Group I. Group II comprised sequences where the expectation value for the best-matching genome was at least 10 orders of magnitude smaller than the best-matching genome from a different class. Group III assignments are made when the NB likelihood score for the best-matching genome is at least 1.5 times greater than the NB likelihood for the best-matching genome from another class. Group IV assignments are based only on the best NB likelihood value. Accuracy of classifications increases with longer contigs [51], so only contigs at least 1000 nucleotides in length were used.

Sequences were functionally annotated through a BLASTP (version 2.2.23) [54] protein similarity search. Annotations were based on the top hit to a reference data set of microbial proteins from the NCBI Protein Clusters database [55] with a 60 % alignment length of the predicted protein with the reference sequence, an expectation value of 10^−5^ or smaller, and neither the predicted protein or reference sequence greater than 1.2 times the length of the other. Additional annotations for enzymes were assigned using a publicly available version (58.1) of the KEGG database [56]. A subset of KEGG pathways and their enzymes (see Table 1 and Additional file 2) related to EBPR metabolism during anaerobic and aerobic cycling, carbon feed source, and nitrogen metabolism were subjected to detailed analysis and were annotated with a more recent version (67.1) of KEGG: butanoate metabolism (BM) for EBPR PHA metabolism, citric acid cycle (CAC), glycolysis/gluconeogenesis (GG), pentose phosphate pathway (PPP), propanoate metabolism (PM) for EBPR propionate metabolism (propionate is the propanoate ion), and nitrogen metabolism (NM).

**Table 1:** List of enzymes by Enzyme Commission number, and common name in text

Enzyme Commission Number	Name
1.1.1.1	alcohol dehydrogenase
1.2.1.12	glyceraldehyde-3-phosphate dehydrogenase
1.6.5.3	NADH:ubiquinone reductase
1.9.3.1	cytochrome-c oxidase
2.3.1.9	acetyl-CoA C-acetyltransferase
2.7.1.11	6-phosphofructokinase
2.7.1.2	glucokinase
2.7.1.63	polyphosphate-glucose phosphotransferase
2.7.2.3	phosphoglycerate kinase
4.2.1.11	phosphopyruvate hydratase
4.2.1.17	enoyl-CoA hydratase
5.4.2.1	phosphoglycerate mutase
6.3.5.4	asparagine synthase

### Identification of putative LGT events

Sequenced reference genomes are typically used for the identification of LGT, but metagenomes rarely produce reliable complete genome sequences. The complete genome of CAP Clade IIA strain UW-1 was however reconstructed from the USA EBPR metagenome. We used this genome to look for initial evidence of LGT in this EBPR community. We performed homology searches, using BLAST, of its genome against itself and 2773 reference genomes, and MGEs used in the EBPR-MGE homology searches. The top hits with a minimum of 60 % shared alignment were used as evidence of potential LGT.

We used two complementary approaches to identify putative LGT events in the EBPR metagenomes. The first approach identified strong disagreement between taxonomic classifications (“classification discordance”) of entire contigs and individual genes within those contigs. The second approach considered incongruence in phylogenetic trees as evidence of LGT. LGT identified by the two approaches were then filtered by homology with known MGEs.

Poor assembly could lead to chimeric contigs and spurious LGT inference. To assess the possible effects of misassembly, we examined the quality of the assemblies from which LGTs were inferred. Reads are available for the AU and USA communities (http://genome.jgi.doe.gov/OZEBPRsludge/OZEBPRsludge.download.htm, projectID=201007), but not the DK community, and none of the three communities had explicit mappings of reads to contigs. We mapped reads back to contigs using a BLASTN search (version 2.2.23), where 70 % of a read was required to align to a contig with a maximum expectation value of 10^−30^, and searched for reads that spanned some or all of the putatively transferred ORF and its neighbors.

### Classification discordance

The taxonomic classification of a whole contig suggests the lineage of the organism from which it was sequenced, but individual protein-coding open reading frames (ORFs) from the contig may differ in their taxonomic assignments. Such disagreements can suggest LGT events with an implied direction of transfer; the donor is the classification of the ORF, and the recipient is the classification of the entire contig. Each predicted ORF was classified at the class level using RITA with the same command-line parameters used above for the contig classifications, with ORFs from group I and group II RITA classifications considered as accurate.

Spuriously classified ORFs originating from classified contigs meeting our length requirements would lead to a questionable inference of LGT. To prevent this, we filtered out candidate transferred ORFs whose best composition-based prediction (i.e., the Naïve Bayes likelihood score) was not at least 15 % better than the contig prediction. If this criterion was satisfied, then the contig was considered a transfer recipient of the implicated ORF.

### Phylogenetic incongruence

Phylogenetic methods incorporate models of the evolutionary process, providing a more accurate representation of evolutionary relationships amongst homologous sequences. We first performed all-versus-all BLAST (version 2.2.23) searches within each community to identify clusters of putative homologous proteins. These sets were then compared with 1642 reference prokaryotic genomes to expand and join clusters. Clusters were represented as an undirected graph using the “networkx” Python package (1.8.1). In the network, a node represents each sequence, and an undirected edge represents a homologous relationship between two sequences. For an edge to be drawn between two EBPR proteins, they must have 70 % sequence identity, and share 60 % alignment length with an e-value of 10^−5^ or smaller. This network was expanded by drawing edges between the nodes, the reference genome sequences and EBPR homologs meeting the BLAST similarity requirements. The network was then split into connected components, or a set of nodes that are connected to each other by a path of edges, where each connected component is considered a cluster.

The resulting clusters were often very large (≥1000 sequences), and included distantly related proteins of little use to LGT inference. To obtain sub-clusters, we constructed phylogenies and extracted subtrees. Sequence alignments were constructed from large clusters using MUSCLE (version 3.8.31) [57] with default settings, and trees were constructed using FastTree (version 2.1.4) [58] with the WAG model of amino acid evolution [59]. We then manually extracted subtrees where FastTree Shimodaira-Hasegawa (SH)-test-based [60, 61] branch support values of at least 70 % denoted clusters of closely related sequences. Subtree extraction, alignment and phylogeny construction was repeated until subtrees comprised a maximum of approximately 200 sequences.

For detecting LGT, phylogenies are typically compared against a reference species tree (e.g. [24]). However, because EBPR community structure can vary over time (e.g. [62]) and metagenomes can represent incomplete samples of the total genetic material [63], crucial taxa including donors of genetic material may not be present in the sample. We used the DendroPy library [64] to calculate the patristic (branch-length) distances between sequences in the same phylogenetic tree, finding for each EBPR sequence the closest EBPR sequence from the same community and the closest reference sequence with an absolute branch length of 0.3 substitutions per site. Sequences with shorter branch lengths should be closest relatives.

### Identifying candidate mobile genetic elements

Potential LGTs from each of the phylogenetic incongruence and classification discordance methods were then filtered by sequences that have homologs, as identified using BLAST with a maximum e-value of 10^−30^ against our custom database of MGEs. We included EBPR sequences with hits to MGE sequences of a different taxonomic class from the EBPR sequence, and had an alignment length of at least 60 % of the query EBPR sequence and 60 % of the subject MGE sequence.

## Results

The published CAP genome was used to find evidence of recent LGT, possibly in the context of the EBPR community. Of the 4562 sequences in the CAP genome, 1438 sequences had hits to genomes outside the Betaproteobacteria with the same e-value as the top CAP hit, suggesting the acquisition of many genes by CAP. The high degree of similarity indicates the possibility that many of these transfers occurred very recently. The observation of these recent transfers led us to search for LGT events in all sampled EBPR community members.

Filtering out contigs that were less than 1000 nucleotides in length reduced the size of the Sanger-sequenced datasets to ~48 % (USA) and ~65 % (AU) of their original sizes, while the Illumina-sequenced DK reactor metagenome was reduced to only ~6 % (Table 2). This result should be expected given the differences in read length and the expected differences in diversity between lab-scale reactors and full-scale reactors [11, 19]. The DK community had the largest number of taxonomic classes represented in the filtered contigs (63), followed by AU (53) and USA (39; see Additional file 3). For all communities, RITA classification Groups I-III accounted for the vast majority of classifications, although the relative proportion of contigs assigned to these groups varied (see Additional file 4). The number of potentially transferred ORFs from the retained contigs also varied by community, analysis type, and the six energy-related pathways.

**Table 2:** Summary of sequences used in analyses from all communities

	USA	AU	DK
# (%) of contigs retained	7,610 (47.96 %)	7,331 (65.52 %)	18,024 (6.69 %)
# (%) of ORFs retained	22,894 (66.06 %)	25,003 (81.15 %)	30,516 (10.14 %)
# enzymes in energy pathways (%) of annotated enzymes	645 (22.13 %)	714 (22.60 %)	524 (22.40 %)

Number of retained contigs, open reading frames from retained contigs, and energy pathway related enzymes (butanoate metabolism, citric acid cycle, glycolysis and gluconeogenesis, nitrogen metabolism, pentose phosphate pathway, and propanoate metabolism) from open reading frames annotated as enzymes. Contigs at least 1000 nucleotides in length were retained

### Classification discordance

Our first approach to identify putative LGT compared the taxonomic classification of an entire contig with the classification of its predicted ORFs. Of the ORFs that had hits to the metabolic pathways of interest, at least 50 % from each community (US: 20 ORFs, 68.9 %, AU: 88 ORFs, 55.7 %, and DK: 58 ORFs, 54.2 %) satisfied the criteria for discordance. All LGTs suggested by this method had hits to annotated MGEs from our database. The number of inferred transfers, the implicated enzymes and the participating taxonomic groups vary among metabolic pathways and communities (Additional file 5). However, some members appear to be more common recipients or donors of gene transfer in all communities and metabolic pathways, with Betaproteobacteria to Gammaproteobacteria (21 transfers) in AU the most common direction of transfer (Additional file 6). LGT events with Alphaproteobacteria as donor and Betaproteobacteria as recipient were the only pattern identified in all three communities.

**Fig. 1:**
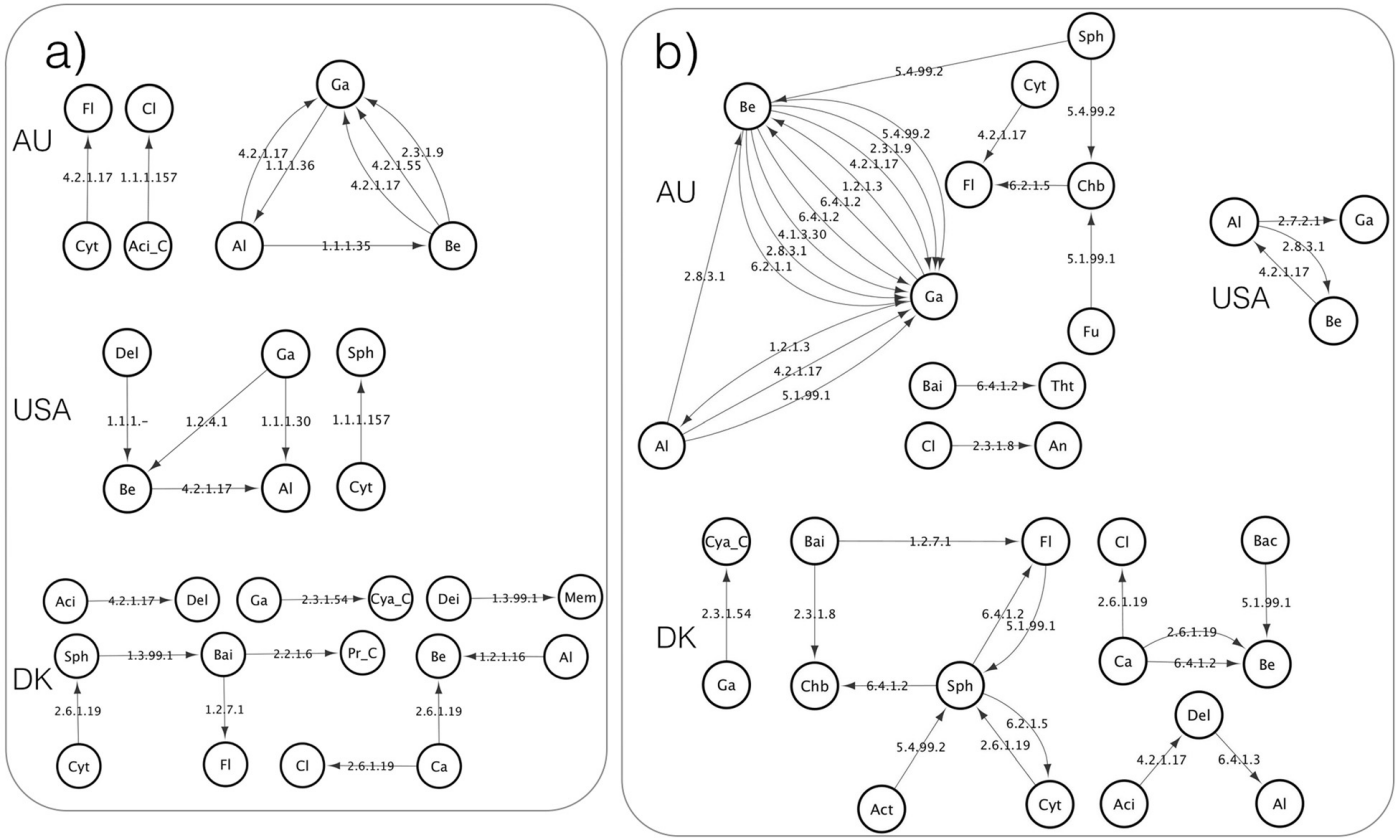
Directed network of gene transfer for Butanoate metabolism and Propanoate metabolism. Directed transfer of enzymes involved in KEGG a) Butanoate metabolism and b) Propanoate metabolism for the Denmark (DK), Australia (AU) and United States (USA) EBPR communities. Taxonomic groups are nodes, and direction of transfer from donor to recipient is indicated by arrows. See Additional file 18 for taxonomic abbreviation guide

Of the six pathways, the pentose phosphate pathway is the only pathway to not have any detected transfers in the DK community (Additional file 5), most likely due to lack of annotated enzymes. Certain pathways have enzymes that appear to have been transferred in all three communities: butanoate metabolism (enoyl-CoA hydratase: EC 4.2.1.17), glycolysis and gluconeogenesis (glucokinase: EC 2.7.1.2), nitrogen metabolism (asparagine synthase: EC 6.3.5.4, cytochrome-c oxidase: EC 1.9.3.1) and propanoate metabolism (EC 4.2.1.17). For example, for butanoate metabolism and propanoate metabolism, enzyme 4.2.1.17 is commonly transferred across all three communities, with directed networks suggesting transfers from Alphaproteobacteria and Betaproteobacteria to Gammaproteobacteria in the AU community, from Betaproteobacteria to Alphaproteobacteria in the USA community, and from Acidobacteria to Deltaproteobacteria (Fig. 1). These genetic exchange networks suggest that PAOs (e.g. Betaproteobacteria) and competing glycogen accumulating organisms (GAOs) (e.g., from Gammaproteobacteria and Alphaproteobacteria) may be involved in transfers of core metabolic enzymes. There also appears to be parallel transfer of genes between taxonomic groups across communities. For example, in glycolysis and gluconeogenesis, 6-phosphofructokinase (EC 2.7.1.11) shows evidence of transfer from Chloroflexi to the Betaproteobacteria in the USA and AU, but not in DK.

**Fig. 2:**
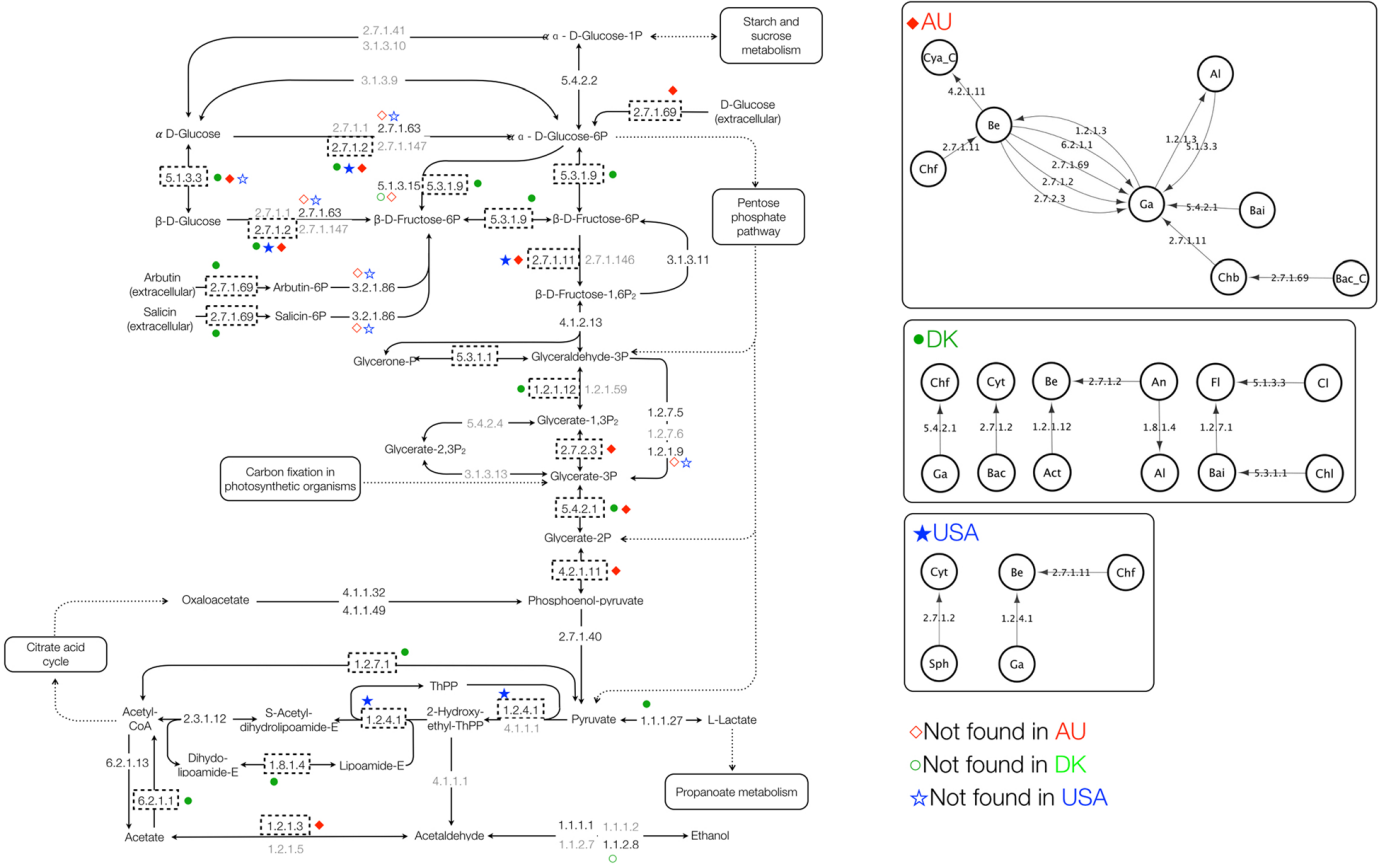
Directed network of gene transfer for the Glycolysis/Gluconeogenesis metabolic pathway. KEGG Glycolysis/Gluconeogenesis metabolic pathway and directed LGT for the Denmark (DK), Australia (AU) and United States (USA) EBPR communities. Dashed boxes indicate LGT, with solid symbols indicating LGT predicted within a community, and hollow symbols indicating enzymes not inferred to be present in a community. EC numbers in gray correspond to enzymes not found in any community. See Additional file 18 for taxonomic abbreviation guide

Transfers may be localized at key in some pathways, for example, where alternative paths between certain metabolites are not present, suggesting an important role for the transfer in the metabolism of the recipients. For example, in glycolysis and gluconeogenesis, glyceraldehyde-3-phosphate dehydrogenase (EC 1.2.1.12) and phosphoglycerate mutase (EC 5.4.2.1) are transferred in the DK community, and phosphoglycerate kinase (EC 2.7.2.3), phosphoglycerate mutase (EC 5.4.2.1) and phosphopyruvate hydratase (EC 4.2.1.11) are transferred in the AU community (Fig. 2). These enzymes are involved in a single path for reactions leading from glyceraldehyde-3-phosphate to phosphoenolpyruvate. Missing enzymes in pathways would increase the need for other enzymes to catalyze key reactions. LGT is one way that genes can be acquired by organisms that need specific enzymes for reactions in pathways. In gluconeogenesis and glycolysis, for example, polyphosphate glucokinase (EC 2.7.1.63) is missing in the AU and USA communities, but glucokinase (2.7.1.2) also catalyzes the reaction ß-D-Glucose to ß-D-Fructose-6-phosphate and shows evidence of LGT in all three communities (Fig. 2). Figures for the other five pathways, indicating gene transfers and the direction of transfer can be found in Additional files 7, 8, 9, 10, 11, 12, 13, 14 and 15.

**Fig. 3:**
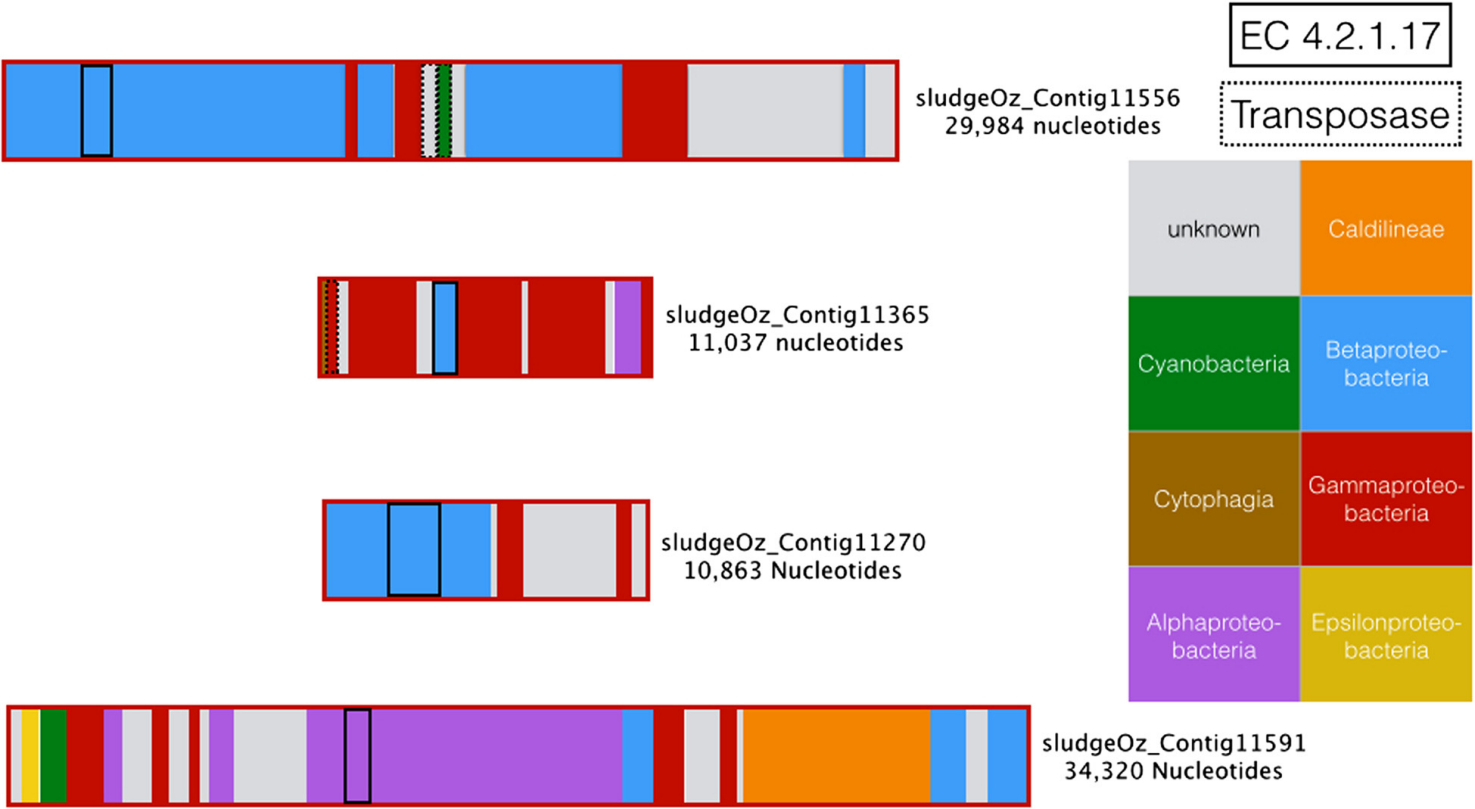
Sample contigs from classification discordance. AU contigs classified as having gammaproteobacterial origin but with an inferred transfer of Enoyl-CoA hydratase (EC 4.2.1.17), an enzyme involved in butanoate metabolism and propanoate metabolism, originating from the Betaproteobacteria or Alphaproteobacteria. Transposases are present on two contigs. Colours represent the taxonomic origin of different genes on each contig according to RITA’s naïve Bayes compositional classifier

Closer scrutiny of the transfers in the directed networks suggests multiple class-level transfers of the same enzyme between specific taxonomic groups. For example, on long contigs, for transfers to the Gammaproteobacteria in the AU community, enoyl-CoA hydratase (EC 4.2.1.17) has been identified as transferred once from the Alphaprotebacteria to the Gammaproteobacteria, and three times from the Betaproteobacteria to three different Gammaproteobacterial contigs. Inspecting the genes on the contig reveals two transposases on one contig and a single transposase on the other (Fig. 3). Classification of sequences in each contig indicates a mixed taxonomic history, suggesting that the present distribution of genes has arisen from a series of independent LGT events.

### Phylogenetic Incongruence

A set of 987 trees covering 46,031 proteins from 1622 reference organisms were extracted from an initial set of 981 trees covering 243,031 proteins from 1642 reference organisms. The direction of transfer is difficult to infer as metagenomic sequencing and quality-filtering approaches remove possible within-community donor and recipient lineages, and tree topologies often cannot distinguish which of two implicated lineages is the most likely donor. A total of 14, 27, and 1 (DK, AU, and USA communities, respectively) predicted EBPR proteins differed from a reference sequence or an EBPR protein of a different taxonomic class by less than 0.3 substitutions per site (Additional file 16). This represented 4.71 %, 8.84 %, and 0.65 % (DK, AU, USA communities, respectively) of all sequences whose closest relative was a member of a different taxonomic class, but did not meet the 0.3 substitutions per site branch length cutoff.

The recipient of the single proposed transfer within the USA community is classified as Gammaproteobacteria, with predicted function associated with glycolysis and gluconeogenesis (EC 1.1.1.1). The AU community accounted for the majority of transfers, with some transfers identified on the same contig, but not evenly distributed across each metabolic pathway. The DK community had the largest number of inferred transfers in the citric acid cycle and nitrogen metabolism pathway. In the AU community, transfers consistently involved sequences belonging to contigs classified as Gammaproteobacteria and Betaproteobacteria, with Alphaproteobacteria, Bacilli and Chlorobia also implicated in transfer of some of the metabolic pathways. For the DK community, no common taxonomic groups were shared across metabolic pathways, and no sequences identified as transferred were classified as Betaproteobacteria. The Cytophagia were implicated in three pathways (butanoate metabolism, citric acid cycle and nitrogen metabolism), while a mixture of the Alphaproteobacteria, Bacteroidia, Flavobacteriia, Gammaproteobacteria, Methanomicrobia, Sphingobacteria are other classes present in the other three pathways (gluconeogenesis and glycolysis, pentose phosphate pathway, propanoate metabolism).

### MGE homology and a common prediction set

Each method of LGT detection differs in its ability to identify different types of LGT events. All high-confidence LGT events have homology with sequences in known MGEs. A substantial number of sequences from each community had hits to known MGEs: 11,718 of 30,516 sequences from the DK community, 16,156 of 24,956 sequences from the AU community, and 15,530 of 22,662 sequences from the USA community. Of those MGE homologs, 2097 DK, 824 AU, and 875 USA community sequences are enzymes in KEGG pathways (Additional file 17).

**Fig. 4:**
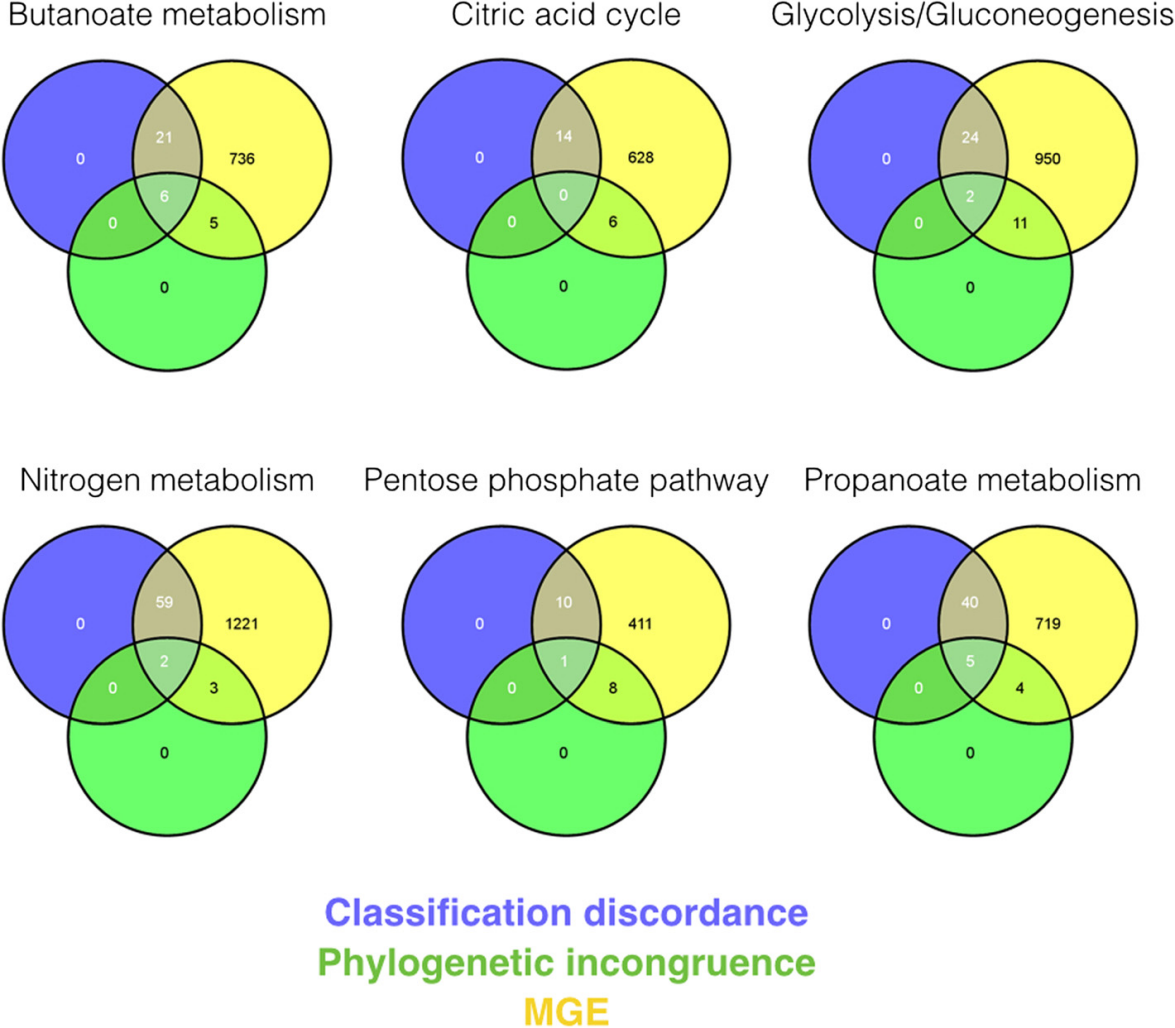
Three-way Venn diagram between classification discordance, phylogenetic incongruence and MGE homology filtering for all sequences from all KEGG pathways. Intersections for circles are the number of transferred genes shared between analyses. Remaining genes not in intersections are unique potential LGT events identified by each analysis. All sequences have homologs with known MGEs. Venn diagrams were generated using Venny (http://bioinfogp.cnb.csic.es/tools/venny/)

Given the very high proportion of metagenomic sequences matching to MGEs, we used additional criteria to support inferences of LGT. To obtain a high-confidence set of transfers, we examined the intersection of the two analyses for each of the six pathways (Fig. 4). Pathways differed by the percent of shared transfers, with each detection method sharing a different percentage of transfers. Up to 55 % of LGT events predicted by the classification discordance approach were shared with the phylogenetic approach. This wide variation in shared LGT events is not correlated to the number of detected LGT events, and illustrates the tendency of each approach to find different types of transfers.

A total of ten sequences, representing five enzymes, were identified as putatively transferred by the two approaches: enoyl-CoA hydratase (EC 4.2.1.17), acetyl-CoA C-acetyltransferase (EC 2.3.1.9), cytochrome-c oxidase (EC 1.9.3.1), phosphoglycerate kinase (EC 2.7.2.3), and 6-phosphofructokinase (EC 2.7.1.11). Of those ten sequences, eight were identified in the AU community, two in the DK community, and none in the USA community. All of the identified enzymes were present on plasmids in the ACLAME database, suggesting a possible mode of transfer. Both analyses almost always identified the same taxonomic classes as donors or as the top hit. The only transfer in DK, enzyme 1.9.1.3, was associated with nitrogen metabolism. Enzyme 2.7.2.3 was unique to GM. Two enzymes, 4.2.1.17 and 2.3.1.9, are common to BM and PM while 2.7.1.11 is common to glycolysis and gluconeogenesis and the pentose phosphate pathway. No common transfers were found that belonged to the CAC. For AU, six of the eight recipient contigs were classified as Gammaproteobacteria, with the remainder Betaproteobacteria and Chlorobia. For DK, the recipient contigs were classified as Bacilli and Cytophagia.

Closer inspection of the contigs that contained the transfers from each analysis provides further support for these putative LGT events. In total, ten of 88 contigs from the AU community, none of the 55 from the DK community, and three of the 19 from the USA community had integrases or transposases on contigs that contained transferred genes from both the classification discordance and phylogenetic incongruence methods. This subset of contigs with integrases and transposases are about two times (AU) or three times (USA) longer, and contain more genes: two to three (AU) or three (USA) more than all contigs with LGTs (Table 3). The relationship between LGT detection and contig length does indicate that longer contigs are more suitable for identification of LGT, and aid the identification of transposases and integrases. This could explain why the DK community did not have any identified transposases and integrases on contigs with an LGT: the majority of contigs were likely too short.

**Table 3:** Length statistics for contigs with putative LGT events

Community	Average Contig Length	Average Number of Genes
DK all	1808.84	1.26
DK LGT six pathways	2885.32	1.91
DK LGT transposases & integrases 6 pathways	__	__
AU all	2883.52	2.70
AU LGT six pathways	11793.48	9.80
AU LGT transposases & integrases 6 pathways	25755.10	23.20
USA all	2477.62	2.37
USA LGT six pathways	23000.37	17.21
USA LGT transposases & integrases 6 pathways	68048.67	53.0

Predicted gene counts and length for each contig from each community that are at least 1000 nucleotides in length, that have a detected LGT event, and those that have an annotated transposase and integrase

Since DK reads were not available and USA LGTs were not part of the shared set of transfers from both analyses, we were only able to assess coverage of AU LGTs. Only seven of the eight AU LGTs had matching reads, but all homologous reads had an expectation value of 0.0. Of the 57 reads with at least partial homology to the putatively transferred ORFs, 50 had alignments that extended into adjacent ORFs, suggesting that the inferred events were not due to misassembly. Two putatively transferred ORFs each had an aligned read that spanned the full length of the ORF (Additional file 18). One LGT had two reads that did not extend into neighbouring ORFs, and started or ended in intergenic regions. Alignments for the remaining five reads partially covered the putatively transferred ORFs.

## Discussion

Using a series of approaches that are applicable to metagenomic data, we found strong evidence that LGT has impacted six energy metabolism pathways in EBPR communities. Some genes appear to have been independently transferred in more than one community. Although some groups are associated with multiple LGT events, no clear patterns of donor/recipient partners emerged for all three communities. The common set of transfers between the two analyses, and MGE homology filtering, provide the strongest evidence for LGT. The majority of transfers shared by both analyses were identified in the AU community, none in the USA community, and only two genes transferred in the DK community, which were the only shared transfers identified in nitrogen metabolism. Differences in predicted events across the three communities may represent independent evolutionary trajectories, differences in local community composition, or biases in observation due to incomplete sampling of the metagenome.

Our contig length and ORF taxonomic quality-filtering approaches favored the detection of a relatively small set of high-confidence LGT predictions. Although choosing the class level decreases the number of potential LGTs found and precludes detection of LGT between members of the same class, the long-range transfers we have identified show the strongest evidence for discordance. Our use of contigs in excess of 1000 nucleotides long considerably reduced the proportion of sequences being retained, especially for the DK community, where the average contig length was 504 nucleotides. However, longer contigs are better for detecting LGT (Table 3). This could be due to a higher probability that genes from a different source are found on longer contigs, or inaccurate classification due to short contigs. Additionally, longer contigs were needed to identify transposases and integrases in tandem with our genes of interest.

Mapping of metagenomic reads to contigs validated most of our LGT inferences; however, one putatively transferred ORF in our high-confidence set did not have any matching reads. Accuracy of assemblies, including metagenomic assemblies, depends on sequencing technology and the complexity of communities [65, 66]. Less-complex communities (~10 genomes) have the most accurate assemblies with Sanger sequencing, and complex communities (100+ genomes) have the most accurate assemblies with Illumina sequencing [66]. Regardless of assembly accuracy, it is unclear why this ORF should be present in the assembled contigs, while having no corresponding match in the reads used to generate those contigs.

Different methods of detecting LGT are often biased towards finding certain types of transfer events [43, 44]. Our approaches do not identify transfers at lower taxonomic levels and are biased towards detection of complete genes. Naïve Bayes likelihood ORF filtering should eliminate many dubious classifications, but does not provide any information about the age of the transfer event. The phylogenetic approach provides information about age of transfers, but identified the fewest candidate LGT events. This is because it requires that the donor lineage in the community or a close relative be sampled, and LGT events that do not appreciably distort the tree will not be detected by this approach.

Different EBPR plants have distinct population characteristics [4, 9], with different operational parameters between the sampled EBPR communities, and full-scale plants being more complex and dynamic than lab-scale reactors [10, 11, 19]. All three communities use different carbon feeds: molasses in DK [23], propionate in AU and acetate in USA [16]. Propionate has been shown to be a more desirable carbon source relative to acetate, providing PAOs a selective advantage over competitors, and resulting in a more stable community over time [12, 17, 67, 68]. The propanoate metabolic pathway, which shows different amounts of evidence for LGT between the three communities, with very few transfers in the acetate-fed USA community, and a large number of transfers in the propionate-fed AU community, especially between the Betaproteobacteria and Gammaproteobacteria. The DK community has an intermediate number of transfers, but with more taxonomic groups implicated than the AU community. The taxonomic composition of EBPR communities is known to change over time [62], and with changing carbon sources [12]; this variability may also manifest through gene exchange between constituents of the community.

Focusing on LGT in energy-related metabolic pathways considered relevant to EBPR function provides context to the role of LGT in EBPR communities. LGT events not in the six energy pathways are also likely to be important in EBPR communities, such as phosphate metabolism, bacteriophage resistance, and flocculation/biofilm formation. Future analyses should also focus on other metabolic pathways for insights into alternative metabolism, and in broad functional categories for overall community functional aspects of LGT. Additional sequencing of EBPR communities would provide further insight into whether there are common LGT events.

## Additional files

Additional file 1:
**Count of RITA reference genomes by taxonomic class**. Additional file 2:
**List of enzymes for each KEGG pathway and their names.**
Additional file 3:
**Classes and counts of contigs classified at the class level greater than 1000 nucleotides long for each community.** Classes shared between each community are also listed. Not all classes could be classified to the class level, and are identified by the taxonomic group they are classified to, followed by ‘(class)’. Additional file 4:
**Count of contigs classified under each RITA classification method.** Group 1 is when homology and nucleotide composition both agree. Group 2 is where the USEARCH expectation value for the best-matching genome was at least 10 orders of magnitude smaller than the best-matching genome from a different class. Group 3 assignments are made when the NB likelihood score for the best-matching genome is at least 1.5 times greater than the NB likelihood for the best-matching genome from another class. Group 4 assignments are based only on the best NB likelihood value. Additional file 5:
**Potential LGTs from taxonomic discordance analysis.** Taxonomic discordance analysis (Naïve Bayes filtered and unfiltered) predicted proteins identified as transferred for each pathway, their EC annotation, class-level taxonomic classification of source (ORF) and recipient (contig). Additional file 6:
**Direction of transfers in classification discordance analysis.**
Additional file 7:
**KEGG citrate cycle pathway and directed LGT for the Denmark (DK), Australian (AU) and United States (USA) EBPR communities.** Dashed boxes indicate LGT, with solid symbols indicating LGT predicted within a community, and hollow symbols indicating missing enzymes in a community. Greyed out enzymes are not found in any community. See Table 1 for enzyme names and Additional file 18 for taxonomic abbreviation guide. Additional file 8:
**KEGG propanoate metabolism pathway and directed LGT for the Denmark (DK), Australian (AU) and United States (USA) EBPR communities.** Dashed boxes indicate LGT, with solid symbols indicating LGT predicted within a community, and hollow symbols indicating missing enzymes in a community. Greyed out enzymes are not found in any community. See Table 1 for enzyme names and Additional file 18 for taxonomic abbreviation guide. Additional file 9:
**KEGG pentose phosphate pathway and directed LGT for the Denmark (DK), Australian (AU) and United States (USA) EBPR communities.** Dashed boxes indicate LGT, with solid symbols indicating LGT predicted within a community, and hollow symbols indicating missing enzymes in a community. Greyed out enzymes are not found in any community. See Table 1 for enzyme names and Additional file 18 for taxonomic abbreviation guide. Additional file 10:
**KEGG nitrogen metabolism pathway and directed LGT for the Denmark (DK), Australian (AU) and United States (USA) EBPR communities.** Dashed boxes indicate LGT, with solid symbols indicating LGT predicted within a community, and hollow symbols indicating missing enzymes in a community. Greyed out enzymes are not found in any community. See Table 1 for enzyme names and Additional file 18 for taxonomic abbreviation guide. Additional file 11:
**KEGG partial nitrogen metabolism pathway and directed LGT for the Denmark (DK), Australian (AU) and United States (USA) EBPR communities in the inner membranes of** ***Nitromonas europaea***, **archaea and bacteria.** Dashed boxes indicate LGT, with solid symbols indicating LGT predicted within a community, and hollow symbols indicating missing enzymes in a community. Greyed out enzymes are not found in any community. See Table 1 for enzyme names and Additional file 18 for taxonomic abbreviation guide. Additional file 12:
**KEGG partial nitrogen metabolism pathway and directed LGT for the Denmark (DK), Australian (AU) and United States (USA) EBPR communities in** ***Rhodospirillum rubum***. Dashed boxes indicate LGT, with solid symbols indicating LGT predicted within a community, and hollow symbols indicating missing enzymes in a community. Greyed out enzymes are not found in any community. See Table 1 for enzyme names and Additional file 18 for taxonomic abbreviation guide. Additional file 13:
**KEGG partial nitrogen metabolism pathway and directed LGT for the Denmark (DK), Australian (AU) and United States (USA) EBPR communities in** ***Kuenenia stuttgartiensis***, ***Candidatus*** **Methylomairabilis oxyfera**, ***Klebsiella oxytoca*** **and** ***Synechococcus PCC7942*** **.** Dashed boxes indicate LGT, with solid symbols indicating LGT predicted within a community, and hollow symbols indicating missing enzymes in a community. Greyed out enzymes are not found in any community. See Table 1 for enzyme names and Additional file 18 for taxonomic abbreviation guide. Additional file 14:
**KEGG partial nitrogen metabolism pathway and directed LGT for the Denmark (DK), Australian (AU) and United States (USA) EBPR communities.** Dashed boxes indicate LGT, with solid symbols indicating LGT predicted within a community, and hollow symbols indicating missing enzymes in a community. Greyed out enzymes are not found in any community. See Table 1 for enzyme names and Additional file 18 for taxonomic abbreviation guide. Additional file 15:
**KEGG butanoate metabolism pathway and directed LGT for the Denmark (DK), Australian (AU) and United States (USA) EBPR communities.** Dashed boxes indicate LGT, with solid symbols indicating LGT predicted within a community, and hollow symbols indicating missing enzymes in a community. Greyed out enzymes are not found in any community. See Table 1 for enzyme names and Additional file 18 for taxonomic abbreviation guide. Additional file 16:
**Potential LGT events from phylogenetic analysis.** Phylogenetic analysis predicted proteins identified as transferred for each pathway, their EC annotation, and class-level taxonomic classification of the contig they are located. Additional file 17:
**Sequences from each community with homologs to known mobile genetic elements.** EBPR sequences with homologs to mobile genetic elements for each pathway, their EC annotation, and class-level taxonomic classification of the contig they are located. Additional file 18:
**List of taxonomic classes and their abbreviations.**
