# Supplementary figures and images for: Transfer of energy pathway genes in microbial enhanced biological phosphorus removal communities
Source: BMC Genomics. 2015 Jul 16;16(1):526. doi: 10.1186/s12864-015-1752-5 (PMC4502571; doi:10.1186/s12864-015-1752-5)

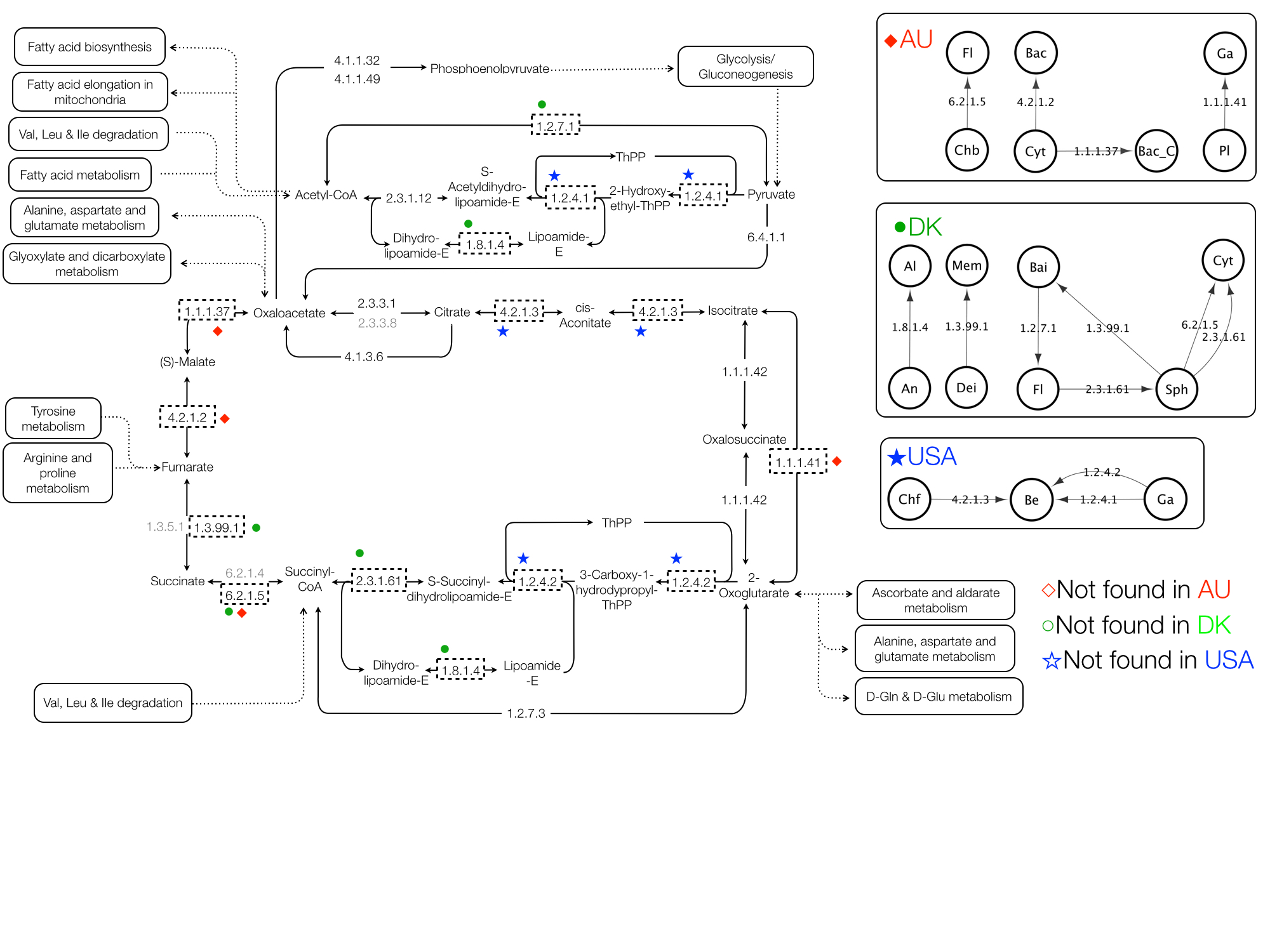

Supplement: Additional file 7: — KEGG citrate cycle pathway and directed LGT for the Denmark (DK), Australian (AU) and United States (USA) EBPR communities. Dashed boxes indicate LGT, with solid symbols indicating LGT predicted within a community, and hollow symbols indicating missing enzymes in a community. Greyed out enzymes are not found in any community. See Table 1 for enzyme names and Additional file 18 for taxonomic abbreviation guide. [file 12864_2015_1752_MOESM7_ESM.jpg]

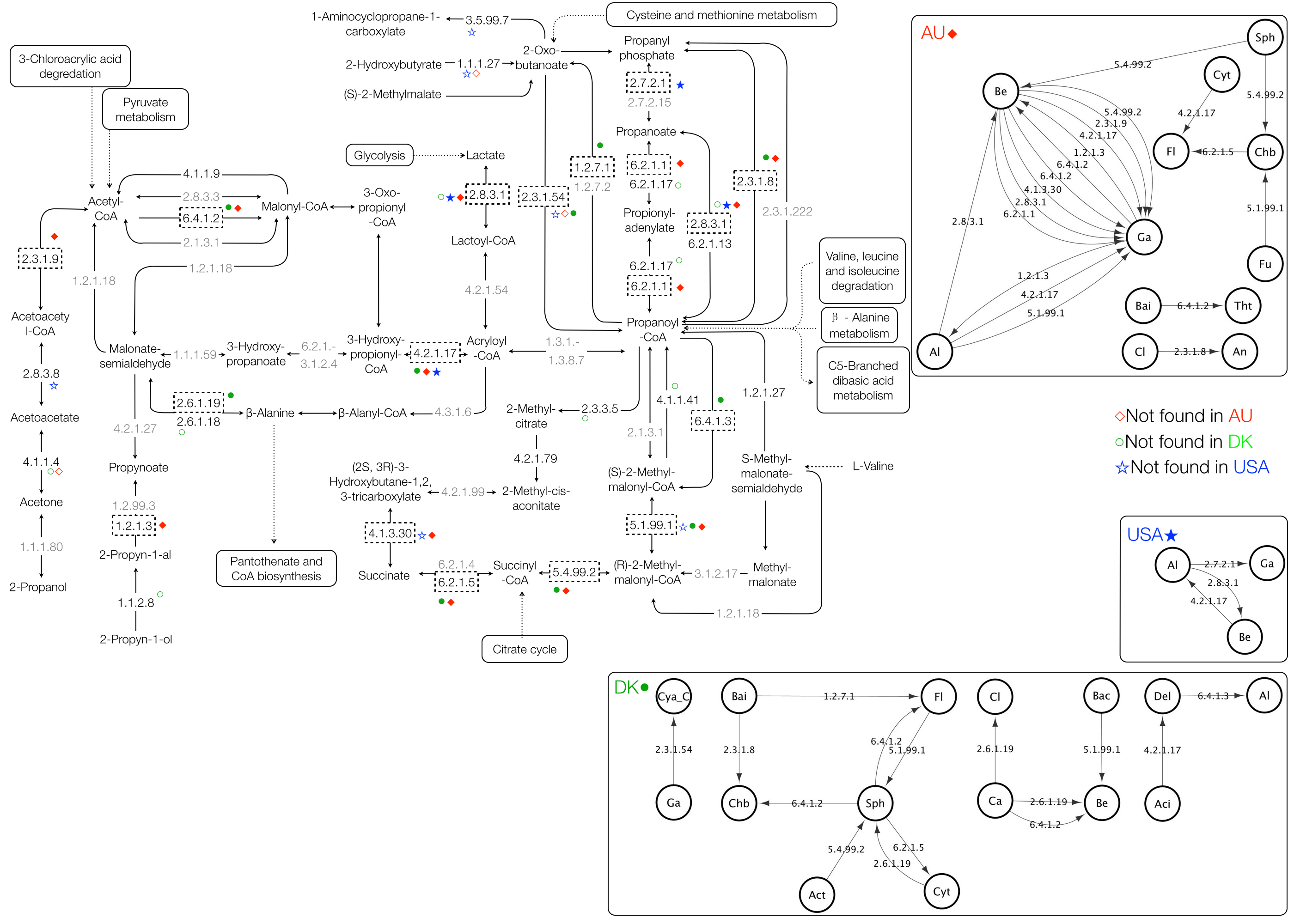

Supplement: Additional file 8: — KEGG propanoate metabolism pathway and directed LGT for the Denmark (DK), Australian (AU) and United States (USA) EBPR communities. Dashed boxes indicate LGT, with solid symbols indicating LGT predicted within a community, and hollow symbols indicating missing enzymes in a community. Greyed out enzymes are not found in any community. See Table 1 for enzyme names and Additional file 18 for taxonomic abbreviation guide. [file 12864_2015_1752_MOESM8_ESM.jpg]

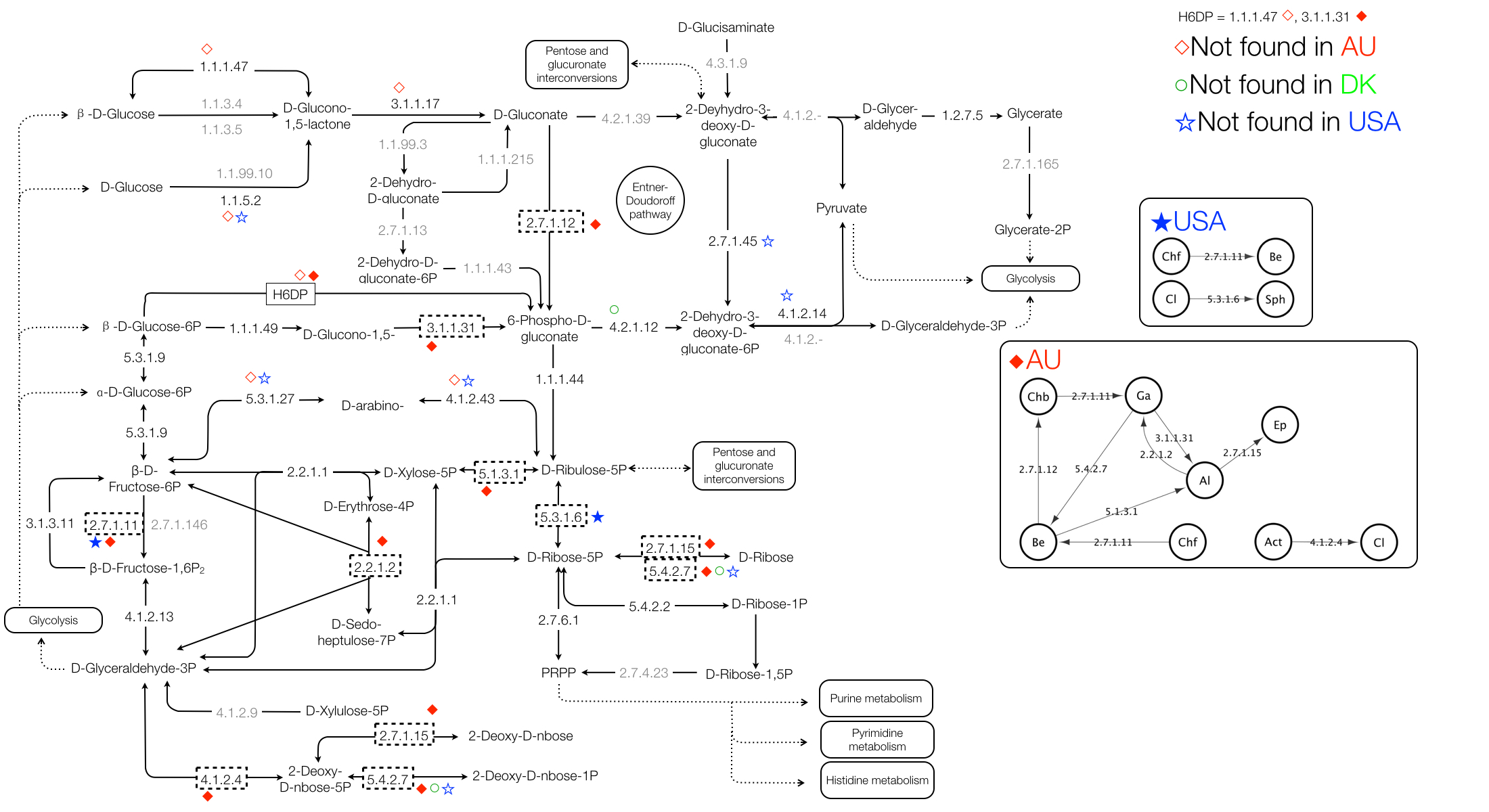

Supplement: Additional file 9: — KEGG pentose phosphate pathway and directed LGT for the Denmark (DK), Australian (AU) and United States (USA) EBPR communities. Dashed boxes indicate LGT, with solid symbols indicating LGT predicted within a community, and hollow symbols indicating missing enzymes in a community. Greyed out enzymes are not found in any community. See Table 1 for enzyme names and Additional file 18 for taxonomic abbreviation guide. [file 12864_2015_1752_MOESM9_ESM.jpg]

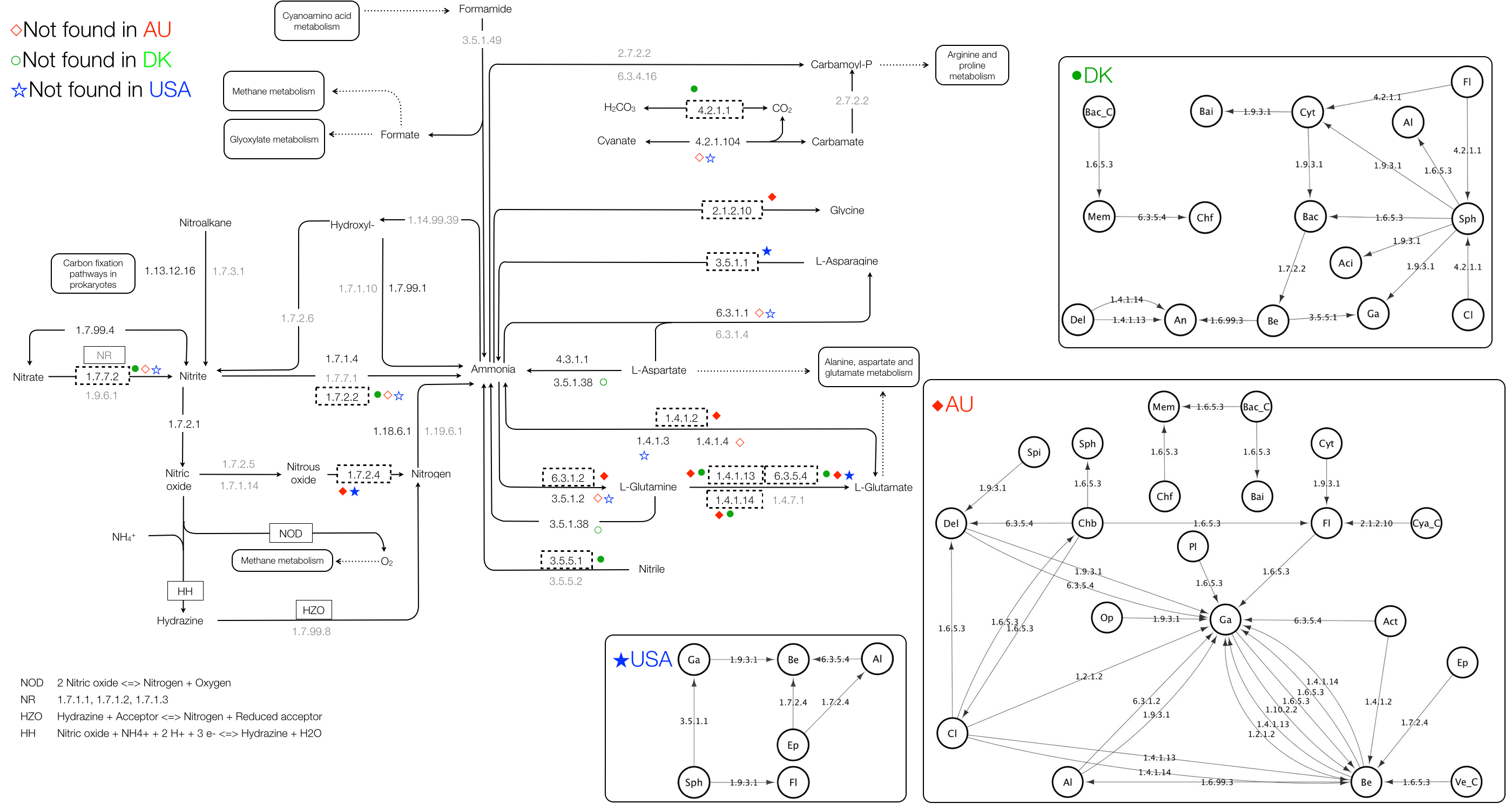

Supplement: Additional file 10: — KEGG nitrogen metabolism pathway and directed LGT for the Denmark (DK), Australian (AU) and United States (USA) EBPR communities. Dashed boxes indicate LGT, with solid symbols indicating LGT predicted within a community, and hollow symbols indicating missing enzymes in a community. Greyed out enzymes are not found in any community. See Table 1 for enzyme names and Additional file 18 for taxonomic abbreviation guide. [file 12864_2015_1752_MOESM10_ESM.jpg]

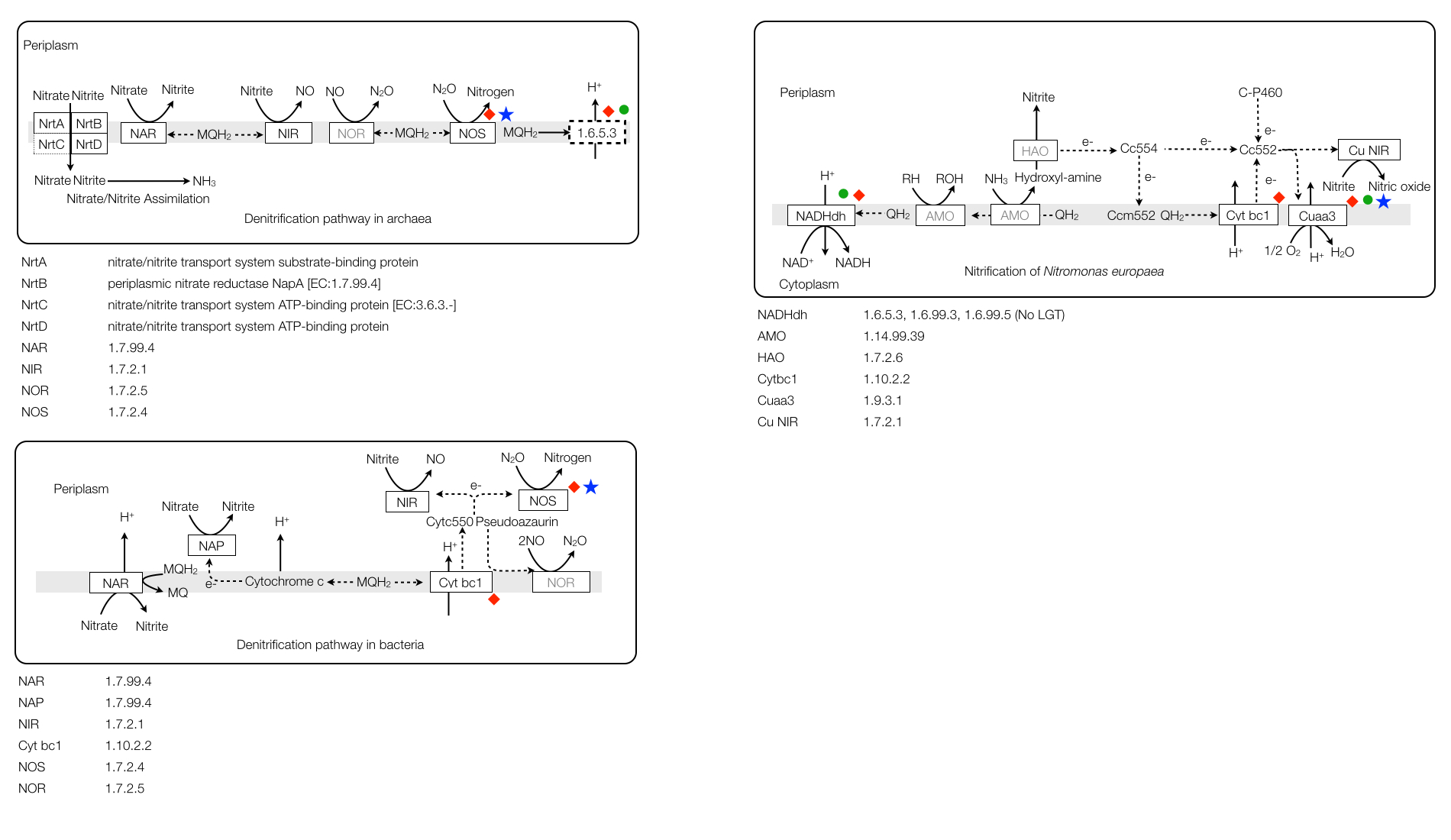

Supplement: Additional file 11: — KEGG partial nitrogen metabolism pathway and directed LGT for the Denmark (DK), Australian (AU) and United States (USA) EBPR communities in the inner membranes of Nitromonas europaea, archaea and bacteria. Dashed boxes indicate LGT, with solid symbols indicating LGT predicted within a community, and hollow symbols indicating missing enzymes in a community. Greyed out enzymes are not found in any community. See Table 1 for enzyme names and Additional file 18 for taxonomic abbreviation guide. [file 12864_2015_1752_MOESM11_ESM.jpg]

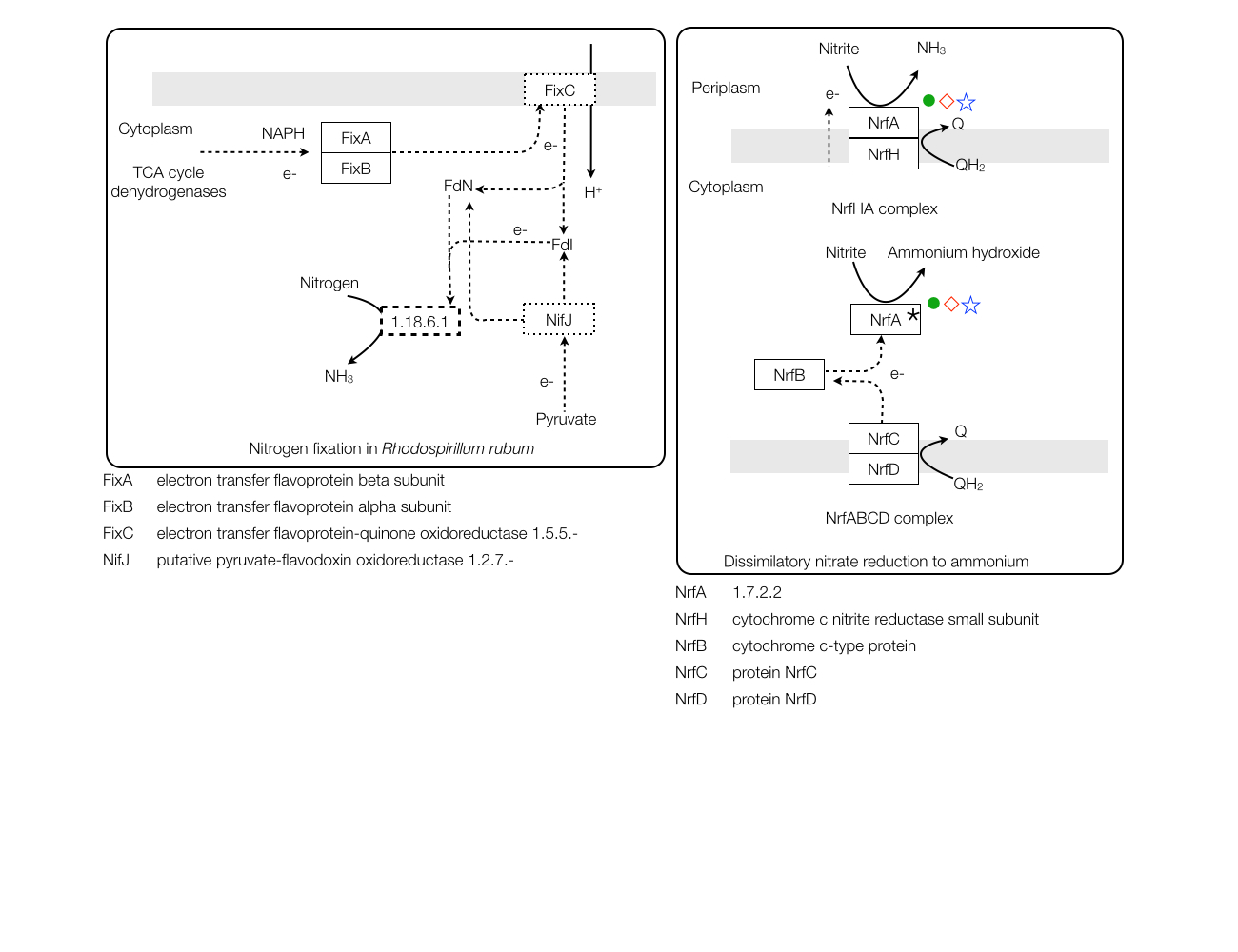

Supplement: Additional file 12: — KEGG partial nitrogen metabolism pathway and directed LGT for the Denmark (DK), Australian (AU) and United States (USA) EBPR communities in Rhodospirillum rubum. Dashed boxes indicate LGT, with solid symbols indicating LGT predicted within a community, and hollow symbols indicating missing enzymes in a community. Greyed out enzymes are not found in any community. See Table 1 for enzyme names and Additional file 18 for taxonomic abbreviation guide. [file 12864_2015_1752_MOESM12_ESM.jpg]

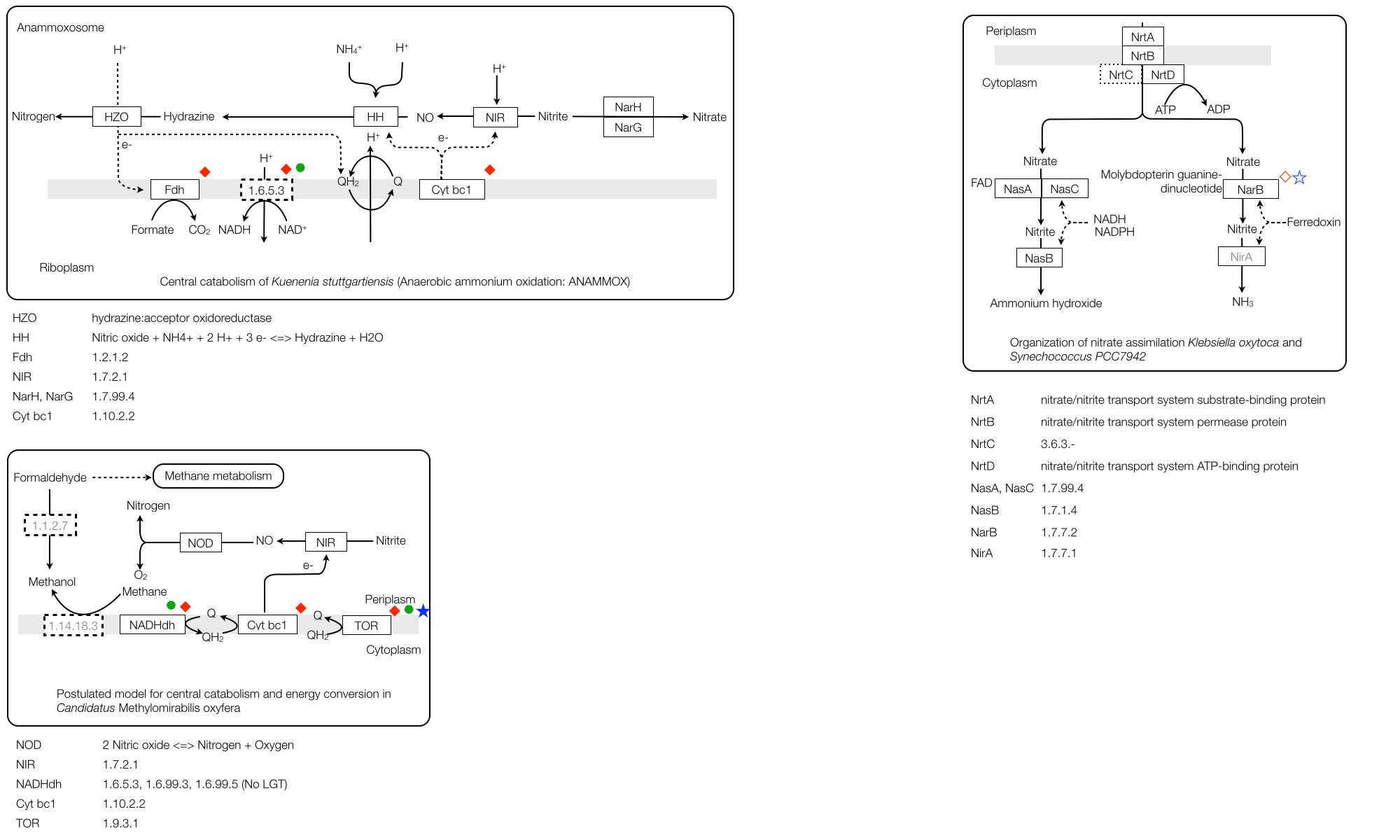

Supplement: Additional file 13: — KEGG partial nitrogen metabolism pathway and directed LGT for the Denmark (DK), Australian (AU) and United States (USA) EBPR communities in Kuenenia stuttgartiensis, Candidatus Methylomairabilis oxyfera, Klebsiella oxytoca and Synechococcus PCC7942 . Dashed boxes indicate LGT, with solid symbols indicating LGT predicted within a community, and hollow symbols indicating missing enzymes in a community. Greyed out enzymes are not found in any community. See Table 1 for enzyme names and Additional file 18 for taxonomic abbreviation guide. [file 12864_2015_1752_MOESM13_ESM.jpg]

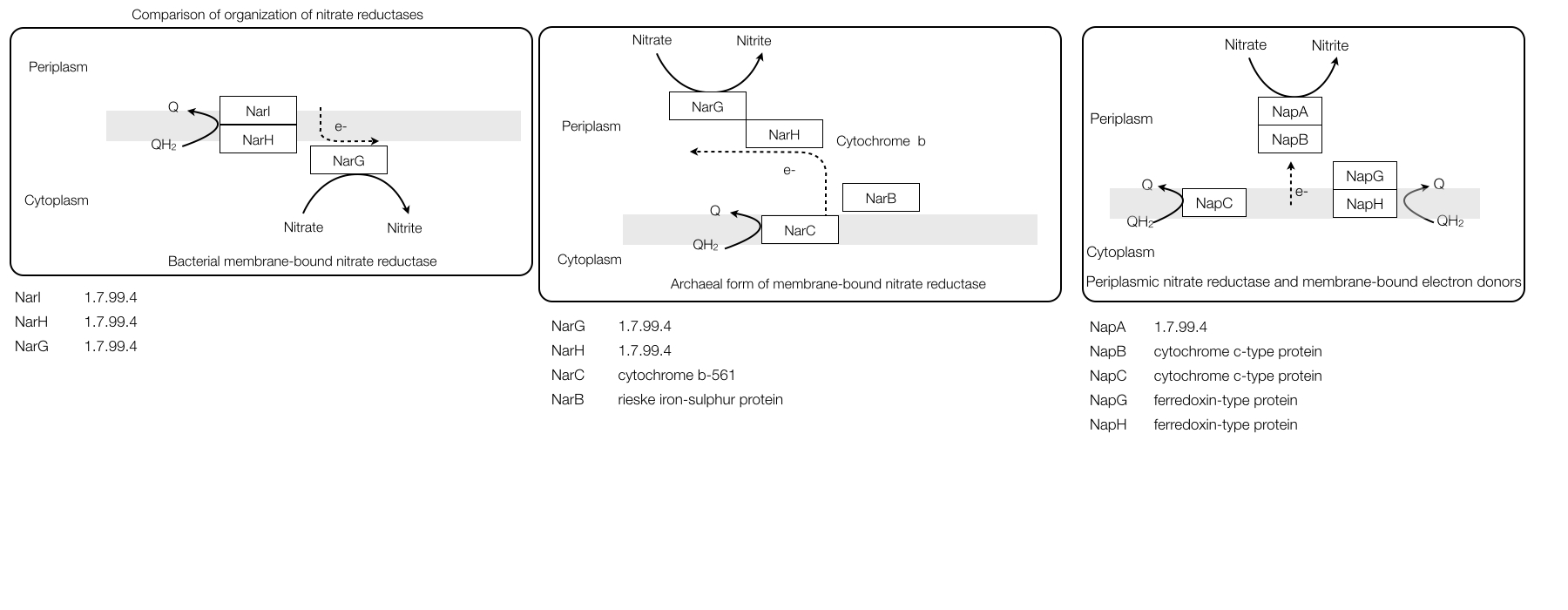

Supplement: Additional file 14: — KEGG partial nitrogen metabolism pathway and directed LGT for the Denmark (DK), Australian (AU) and United States (USA) EBPR communities. Dashed boxes indicate LGT, with solid symbols indicating LGT predicted within a community, and hollow symbols indicating missing enzymes in a community. Greyed out enzymes are not found in any community. See Table 1 for enzyme names and Additional file 18 for taxonomic abbreviation guide. [file 12864_2015_1752_MOESM14_ESM.jpg]

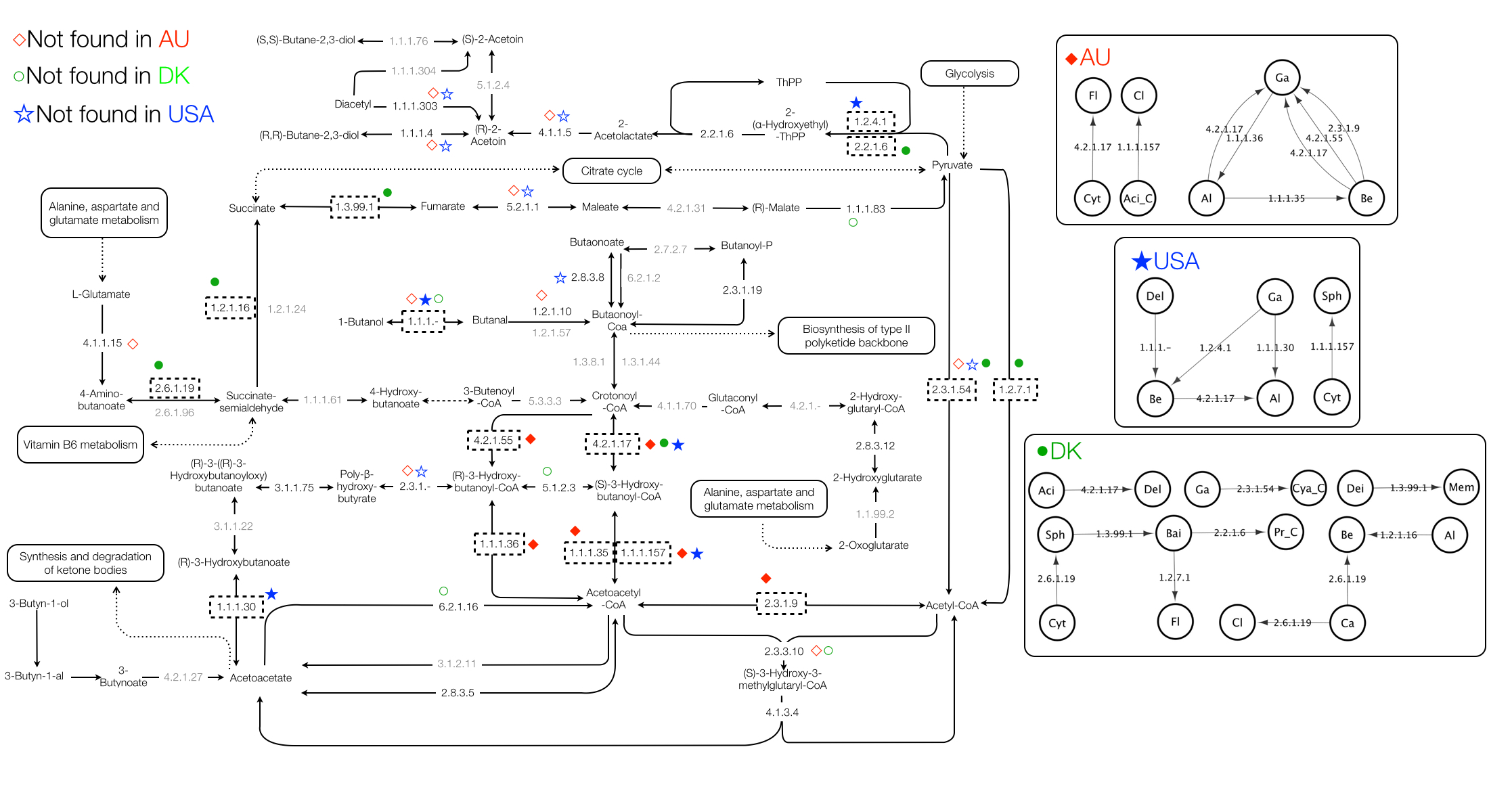

Supplement: Additional file 15: — KEGG butanoate metabolism pathway and directed LGT for the Denmark (DK), Australian (AU) and United States (USA) EBPR communities. Dashed boxes indicate LGT, with solid symbols indicating LGT predicted within a community, and hollow symbols indicating missing enzymes in a community. Greyed out enzymes are not found in any community. See Table 1 for enzyme names and Additional file 18 for taxonomic abbreviation guide. [file 12864_2015_1752_MOESM15_ESM.jpg]
